# TB-ML—a framework for comparing machine learning approaches to predict drug resistance of *Mycobacterium tuberculosis*

**DOI:** 10.1093/bioadv/vbad040

**Published:** 2023-03-23

**Authors:** Julian Libiseller-Egger, Linfeng Wang, Wouter Deelder, Susana Campino, Taane G Clark, Jody E Phelan

**Affiliations:** Faculty of Infectious and Tropical Diseases, London School of Hygiene & Tropical Medicine, London WC1E 7HT, UK; Faculty of Infectious and Tropical Diseases, London School of Hygiene & Tropical Medicine, London WC1E 7HT, UK; Faculty of Infectious and Tropical Diseases, London School of Hygiene & Tropical Medicine, London WC1E 7HT, UK; Faculty of Infectious and Tropical Diseases, London School of Hygiene & Tropical Medicine, London WC1E 7HT, UK; Faculty of Infectious and Tropical Diseases, London School of Hygiene & Tropical Medicine, London WC1E 7HT, UK; Faculty of Epidemiology and Population Health, London School of Hygiene & Tropical Medicine, London WC1E 7HT, UK; Faculty of Infectious and Tropical Diseases, London School of Hygiene & Tropical Medicine, London WC1E 7HT, UK

## Abstract

**Motivation:**

Machine learning (ML) has shown impressive performance in predicting antimicrobial resistance (AMR) from sequence data, including for *Mycobacterium tuberculosis*, the causative agent of tuberculosis. However, current ML development and publication practices make it difficult for researchers and clinicians to use, test or reproduce published models.

**Results:**

We packaged a number of published and unpublished ML models for predicting AMR of *M.tuberculosis* into Docker containers. Similarly, the pipelines required for pre-processing genomic data into the formats required by the models were also packaged into separate containers. By following a minimal container I/O standard, we ensured as much interoperability as possible. We also created a command-line application, TB-ML, which can be used to easily combine pre-processing and prediction containers into complete pipelines ready for predicting resistance from novel, raw data with a single command. As long as there is adherence to this minimal standard for the container interface, containers produced by researchers holding new models can likewise be included in these pipelines, making benchmark comparisons of different models simple and facilitating faster uptake in the clinic.

**Availability and implementation:**

TB-ML contains a simple Docker API written in Python and is available at https://github.com/jodyphelan/tb-ml. Example Docker containers for resistance prediction and corresponding data pre-processing as well as a tutorial on how to create new containers for TB-ML are available at https://tb-ml.github.io/tb-ml-containers/.

**Contact:**

jody.phelan@lshtm.ac.uk

## 1 Introduction

The emergence of antimicrobial resistance (AMR) represents a serious threat for global public health with an estimated 5 million deaths associated with drug-resistant bacterial infections in 2019 alone (Murray *et al.*, 2022). For example, *Mycobacterium tuberculosis* bacteria are becoming increasingly resistant to commonly used drugs, making the control of tuberculosis disease problematic, especially in high-burden settings (WHO, 2021). Whole genome sequencing can identify known mutations driving AMR, enabling the genotypic profiling of resistance phenotypes. This profiling has the potential to replace laboratory-based drug susceptibility testing as a means of informing clinical treatment decisions in a more timely manner (Boolchandani *et al.*, 2019). Traditional approaches for such *in silico* resistance profiling, however, rely on databases of known resistance-conferring mutations and, even for well-studied bacteria such as *M.tuberculosis*, not all AMR mutations are known.

Therefore, there has been great interest lately in the application of machine learning (ML) to the problem of resistance prediction (see Kim *et al.*, 2022, for a recent review). In addition to using potentially unknown genomic variants, ML models can also combine information on multiple mutations, allowing them to take epistatic interactions into account. However, the use of ML models also comes with its downsides. One challenging aspect is that some model types are ‘black boxes’ with limited interpretability. Additionally, while superior accuracy compared to traditional approaches has been shown for a number of ML methods (e.g. Kouchaki *et al.*, 2019), large-scale systematic comparisons of published models (e.g. to determine the best model architecture for a certain pathogen or type of genomic input data) are still lacking. One major obstacle in this regard is that researchers rarely publish trained models in a form that can be readily used for generating predictions from new data with little extra effort. Instead, when attempting to reproduce results achieved by an ML model, one often has no other option than to re-train it from scratch. This task usually comes with several practical difficulties, including (but not limited to) unpublished training data, lacking source code (for model training or the pre-processing of raw reads into a format ready for training/prediction) and inadequate information on software dependencies and versions. These issues are likewise impeding quick translation of ML models into the clinic, which is often called for in light of their impressive performance.

## 2 A minimal standard for containerized resistance prediction models

To solve this reproducibility and utilization challenge, we propose that ML models are placed into Docker containers, enabling others to easily predict from new data. We suggest the containerization of any pre-processing code separately from the ML model so that different sources of genomic inputs can be used. To support this, we defined a minimal standard for the interface between pre-processing and prediction containers and provide a Python application (‘TB-ML’) handling inter-container communication. It can be used to combine different containers adhering to this simple standard in a mix-and-match fashion to build prediction pipelines covering the whole prediction process, from raw reads to final report, in a single command.

In order to facilitate the interoperability of containers for pre-processing and prediction while retaining as much flexibility as possible, TB-ML has the following requirements for the container interface: (i) any output that should be added to the final prediction report has to be printed to STDOUT; (ii) output used by other containers needs to be written to files (ideally in CSV format); (iii) prediction containers should only predict and perform no pre-processing tasks. When possible, prediction containers should also accept input in CSV format (e.g. one-hot-encoded sequences or called variants).

## 3 TB-ML functionality and implementation

Containers adhering to the aforementioned rules can be combined into pipelines with our command-line tool TB-ML. In most basic cases, only two containers will be needed to predict resistance from raw input data: one for bioinformatic processing of the inputs into a suitable format (e.g. called variants or one-hot-encoded consensus sequences) and one for prediction. However, pipelines of arbitrary complexity are possible since TB-ML does not impose a limit on the number of containers used. It has been implemented in Python and mostly consists of a basic Docker API to facilitate the launching of the containers and data transfer between them. All steps are run in a temporary directory. The command-line interface only has a single flag (–container). It is used to specify the name of a Docker image which can be followed by a single string holding all the arguments to be passed to the container. This way, the whole pipeline can be specified in a single command.

At https://tb-ml.github.io/tb-ml-containers/, we provide example Docker containers for pre-processing and resistance prediction on *M.tuberculosis* data. So far, they include several neural networks (including one created by Green *et al.*, 2022 and a variation which is independent of the dimensionality of the input data), one random forest model, and four pre-processing pipelines to generate input data for the models from either raw or aligned reads.

## 4 Discussion and conclusions

After showing the great potential of ML in AMR prediction, the maturing field now needs to focus on improving reproducibility, usability, and ease of benchmarking. The framework for combining Docker containers provided here represents a first step in this direction. TB-ML is easily installed, requiring only Python and Docker. The standard for inter-container communication is flexible enough to be readily extended to other prediction methods or pre-processing steps, including non-tuberculosis AMR applications. Further, TB-ML can be included into existing profiling software, e.g. TB-Profiler (Phelan *et al.*, 2019), in the future. Overall, this effort aids those working on the genotypic prediction of AMR in comparing, implementing and interpreting the results from ML models, thereby assisting the personalization of clinical interventions, and ultimately improving infection control.

## Data Availability

There are no new data associated with this article.
